# Individual, Family, and Community Predictors of Overweight and Obesity Among Colombian Children and Adolescents

**DOI:** 10.5888/pcd11.140065

**Published:** 2014-08-07

**Authors:** Ines Gonzalez-Casanova, Olga Lucia Sarmiento, Michael Pratt, Julie A. Gazmararian, Reynaldo Martorell, Solveig A. Cunningham, Aryeh Stein

**Affiliations:** Author Affiliations: Olga Lucia Sarmiento, Facultad de Medicina, Universidad de los Andes, Bogotá, Colombia; Michael Pratt, Rollins School of Public Health, Emory University, Facultad de Medicina, Universidad de los Andes, Bogotá, Colombia, and Centers for Disease Control and Prevention, Atlanta, Georgia; Julie A. Gazmararian, Reynaldo Martorell, Solveig A. Cunningham, Aryeh Stein, Rollins School of Public Health, Emory University, Atlanta, Georgia.

## Abstract

**Introduction:**

Information from high-income countries is often used to design childhood obesity prevention interventions in low- and middle-income countries, even though determinants may differ greatly between settings.

**Methods:**

We examined the associations of individual, family (household), and community (municipality) characteristics with body mass index (BMI) *z* scores and likelihood of overweight among children aged 5 to 18 years measured for the Colombian National Nutrition surveys of 2005 (n = 9,119) and 2010 (n = 21,520). We used 3-level hierarchical linear models with child as level 1, household as level 2, and municipality as level 3.

**Results:**

The prevalence of combined overweight and obesity among Colombian children and adolescents was 15.7% in 2005 and 16.6% in 2010. The household level explained 40% in 2005 and 31% in 2010 of the variability in BMI *z* scores. Wealth was positively associated with BMI in 2005 (0.09 increase in *z* score per wealth quintile) and 2010 (0.13 increase in *z* score per wealth quintile) (*P* < .01). Children and adolescents from extended families had higher BMI *z* scores than those from nuclear families; BMI *z* scores were inversely associated with the number of family members living in the same household. The municipality level explained only between 2% and 3% of the variability in BMI. Income inequality was positively associated with BMI *z* scores in 2010.

**Conclusion:**

These patterns differ from those commonly described in high-income countries and suggest more appropriate opportunities for interventions to prevent child and adolescent obesity in Colombia and other Latin American settings and populations.

## Introduction

Overweight and obesity in children and adolescents are emerging public health concerns in low-, middle-, and high-income countries (1–3) Excess adiposity among youth increases risk of adult obesity, cardiometabolic disease, and psychosocial problems (4). Because children and adolescents are rapidly developing, both physically and cognitively, they may be especially vulnerable to social and community influences (4). Research on the determinants of overweight and obesity has evolved from conceptual models based on simple relationships between individual factors and excessive weight to models identifying complex relationships among individual, social, and environmental factors (5,6). Studies in high-income countries have identified certain family and community factors as determinants of overweight and obesity among children and adolescents (5,7). For instance, elements of family structure (such as parental beliefs), being an only child, certain diet and physical activity behaviors, and socioeconomic factors (such as lack of economic resources or education) have been identified as determinants (8). Living in underserved and poorly designed urban neighborhoods is also associated with higher risk of overweight and obesity (9). Conversely, availability of parks and recreation facilities has been shown to promote physical activity among children (10). In Los Angeles, within-neighborhood inequality seems to protect against increases in the prevalence of overweight and obesity (11). Information on the correlates of overweight and obesity among children and adolescents is useful when designing and targeting policies and interventions (12).

Most countries in Latin America experienced large increases in the prevalence of overweight and obesity during the past 20 years (1–3). Information on the determinants of overweight and obesity in Latin American and other low- and middle-income countries is scarce, particularly for children and adolescents (2); consequently, information from high-income countries is often used to design policies and interventions (13). The objective of this study was to assess the potential influence of individual, family, and community predictors on weight status of Colombian children and adolescents by using multilevel modeling techniques to represent the complex hierarchical relationships.

## Methods

### Study sample

**Figure 1:**
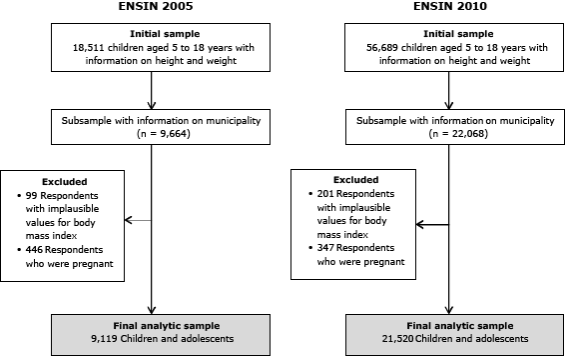
Sample selection from the Colombian National Nutrition Survey (ENSIN) in 2005 and 2010 for the analysis of individual, family, and community predictors of child and adolescent overweight and obesity in Colombia.

We analyzed data from the Encuesta Nacional de la Situacion Nutricional en Colombia (Colombian National Nutrition Survey [ENSIN]) in 2005 (14) and 2010 (15) as part of the Colombian Demographic and Health Surveys (DHS) (16) and from the 2005 Colombia National Census. The samples for the 2 surveys were obtained by using a multistage, stratified, clustered design, and they are representative of the Colombian population at the national and regional levels (14,15). All children and adolescents aged 5 to 18 years with information on height and weight were included. We excluded the following from the final analysis: those with implausible values (defined as more than 6 standard deviations [SDs] beyond the mean of the World Health Organization [WHO] reference population) for weight, height, or body mass index (BMI); those with missing values on any individual, family, or community predictor; and those who were pregnant (Figure 1).

### Contextual model and variables

**Figure 2:**
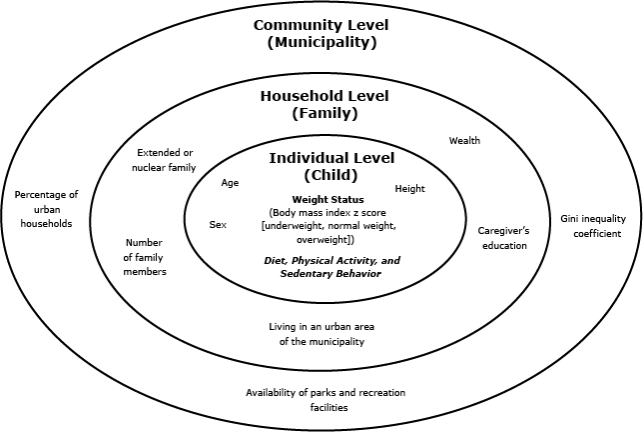
Ecological model of childhood obesity for analyzing individual, family, and community predictors of child and adolescent overweight and obesity in Colombia. This model was adapted from the ecological model of childhood obesity proposed by Devison and Birch (5). The child is situated at the individual level, the family at the household level, and the municipality (administrative unit of Colombia) at the community level. The predictors in the model were based on the original model and on information available from the Colombian National Nutrition Survey or the National Census. Data on diet, physical activity, and sedentary behavior were not included in this study because of lack of data.

Our analysis was based on the contextual model of childhood obesity proposed by Davidson and Birch (5), which has 3 levels — the individual, the family, and the community — and identifies factors and their interactions at each level (6) (Figure 2). Variables were selected according to this model, evidence of the association with childhood obesity, and data availability in 2005 or 2010. 

#### Level 1: Child (individual)

Height and weight were measured directly by trained nutritionists following standard protocols (14,15). We computed BMI, the outcome variable, as weight (in kilograms) divided by height (in meters) squared and BMI *z* score relative to the WHO reference population for children aged 5 to 18 years (17). A BMI *z* score indicates how many units (in SDs) a child’s BMI is above or below the mean BMI value for their age group and sex. For example, a *z* score of 1.2 indicates that a child is 1.2 SDs above the mean value, and a z score of –1.2 indicates a child is 1.2 SDs below the mean value.

WHO defines underweight as a BMI z score greater than 2 SDs below the mean, overweight as a BMI *z* score greater than 1 SD above the mean, and obesity as a BMI z score greater than 2 SDs above the mean. BMI *z* score was used as a continuous dependent variable in linear models and classified into underweight (less than −1 SD [different from the WHO definition]), normal weight (−1SD to 1 SD) and overweight (>1 SD) for multinomial models. Too few (~2%) of the study sample had a BMI z score greater than 2 to permit statistical analysis. Data on age were obtained from information on date of birth and date of interview and coded as integer years. Height was measured to the nearest centimeter. Sex was coded as male or female.

#### Level 2: Family (household)

To measure socioeconomic status, a wealth index was calculated using a principal components analysis that included 50 items (eg, car ownership, television ownership, type of household flooring, presence of a domestic servant). Households were classified into 5 categories based on population quintiles (1st = poorest to 5th = wealthiest) (18).

Households in the main city of the municipal district and in areas of the municipality with basic services such as water, electricity, pavement, and health services were categorized as urban. Households outside the main city and in areas without such services were categorized as rural (19). Living in an urban area of the municipality was included at the household level because in Colombia urbanization varies within municipalities (ie, the level in which variables are included does not affect the distribution of the variability in BMI *z* scores explained by each level).

As a measure of education levels, we used data on the number of years of schooling of the “most likely” caregiver according to traditional gender roles in Colombia (20). The mother was designated as the most likely caregiver unless she was not alive or was not living with the child. In those cases, we used data on years of schooling of the father, grandmother, or grandfather (in that order of preference).

Households with family members besides the parents and their offspring (eg, grandparents, aunts, uncles) were classified as extended family households. Households in which only the parents and their offspring lived were classified as nuclear family households. Information on family members in a household was self-reported by the respondent to the DHS household questionnaire. We also recorded the number of family members living in the same household.

#### Level 3: Community (municipality)

The Gini coefficients for Colombian municipalities were calculated on the basis of information from the 2005 Colombian National Census (19). The Gini coefficient is used to measure inequalities in resources (21); it is scored on a scale of 0 to 100, with 0 representing complete equality and 100 representing complete inequality. Gini coefficients were expressed in this study as *z* scores. Information from the 2005 Columbian National Census was used to determine park density: the number of census units (per municipality) that had parks (19). Park density was expressed as *z* scores. The percentage of urban households per municipality was calculated from the number of urban households and the total number of households in each municipality among respondents to ENSIN 2005 and ENSIN 2010.

### Statistical analysis

An initial descriptive analysis of means of continuous variables and frequencies of categorical variables was conducted. Potential systematic bias was assessed by exploring patterns of missing data. BMI was tested for normality by using standard procedures. Bivariate regressions were conducted to understand the relationships between different predictors in the conceptual model.

Five 3-level hierarchical linear models were developed, each of which included all predictors from each of the 3 levels. The models included 1) an unconditional model with random intercepts and no predictors; 2) a random-intercept fixed-slope model with level 1 predictors only; 3) a random-intercept fixed-slope model with level 2 predictors only; 4) a random-intercept fixed-slope model with level 3 predictors; and 5) a random-intercept fixed-slope model with all predictors at all 3 levels. BMI *z* score was the outcome for all models. The predictors were age and sex at level 1; wealth index, caregiver’s education, percentage of urban households, extended family, and family size at level 2; and Gini coefficient *z* scores, park density *z*-scores, and percentage of urban households at level 3. Significance was declared at *P* < .05. We also tested for cross-level interactions with wealth and retained them when significance was *P* < .05 in either 2005 or 2010. We assessed the multicollinearity of the predictors by using variance-inflation factors. The final model was selected on the basis of theoretical framework and best fit (deviance statistic, Akaike Information Criterion, and Bayesian Information Criterion). The same models were examined by using data from 2010 (except for census data, which are from 2005 only). Intraclass correlation coefficients were computed at level 2 (*r*_0_ */(u*_00_ *+* σ^2^ *))* and level 3 (σ^2^ */(u*_00_ *+* *r*_0_ *))* by dividing the variance for each level by the variances at the other 2 levels. Reliability estimates reflect the ability of each cluster (ie, individual, family, and municipality) to predict itself. The final model was also computed by using multinomial hierarchical linear modeling techniques to explore nonlinear associations of the predictors with BMI *z* score. All statistical analyses were conducted using SAS 9.2 (SAS Institute, Cary, North Carolina), SAS-callable Sudaan, and HLM 7.0 (Scientific Software International, Skokie, Illinois). Weights at the individual level were used to account for the sample design.

## Results

The final sample consisted of 9,119 children, 4,420 households, and 177 municipalities in 2005 and 21,520 children, 12,452 households, and 104 municipalities in 2010. There were 1 to 11 children per household and 20 to 621 households per municipality. The prevalence of combined overweight and obesity in Colombian children and adolescents was 15.7% in 2005 and 16.6% in 2010. BMI was normally distributed in both the 2005 and 2010 samples (Table 1). The means of the individual-level variables (BMI *z* score, age, sex, and height) were consistent across surveys. At the family and municipality level, most variables were consistent across surveys, except for percentage of urban households, which was significantly higher in 2010 than in 2005.

**Table 1:** Descriptive Statistics for the Analysis of Predictors of Overweight and Obesity Among Children and Adolescents, Colombian National Nutrition Surveys, 2005 and 2010^a^

Variable	2005		2010	
	n	Mean (SD)	n	Mean (SD)
**Child (individual)**				
BMI *z* score^b^	9,119	−0.08 (0.99)	21,520	−0.04 (1.01)
Height, cm		139.8 (19.5)		141.1 (19.3)
Height-for-age *z* score^b^		−0.70 (1.02)		−0.69 (1.01)
Age, y		11.2 (3.9)		11.6 (3.8)
Girl, %		52 (50)		50 (50)
**Family (household)**				
Wealth index^c^	4,420	2.8 (1.3)	12,452	3.0 (1.3)
Urban^d^, % of households		70 (46)		82 (39)
Extended family, % of households		49 (50)		44 (50)
Family size, no. of members		5.2 (2.1)		4.7 (1.9)
**Community (municipality)**				
Gini coefficient *z* score	177	−0.13 (0.97)	104	−0.13 (1.07)
Park density *z* score		0.02 (1.21)		−0.01 (1.15)
Percentage urban^e^, mean		60.8 (29.8)		66.5 (25.8)

Abbreviations: SD, standard deviation; BMI, body mass index.

a Sample weights were used to account for survey design.

b Relative to the World Health Organization (WHO) reference population for children aged 5 to18 years, 2007. WHO defines underweight as a BMI *z* score greater than 2 SDs below the mean, overweight as a BMI *z* score greater than 1 SD above the mean, and obesity as a BMI z score greater than 2 SDs above the mean. BMI *z* score was used as a continuous dependent variable in linear models and classified into underweight (less than −1 SD [different from the WHO definition]), normal weight (−1SD to 1 SD) and overweight (>1 SD) for multinomial models.

c Households were classified into 5 quintiles (1st = poorest to 5th = wealthiest).

d A household is classified as urban if it is located in an area of the municipality with basic services, such as electricity, water, a town hall, etc. Otherwise, the household is classified as rural.

e Variable created by dividing the number of urban households in a municipality by the total number of households in the municipality.

### Three-level hierarchical linear model of BMI *z* score

The unconditional models showed reliability estimates of 0.56 in 2005 and 0.42 in 2010 at the household level and 0.46 in 2005 and 0.53 in 2010 at the municipality level (Table 2). These values indicate a significant correlation of BMI *z* score among children living in the same household and among households in the same municipality. The household level explained 40% of the variability in BMI *z* scores in 2005 and 31% in 2010; the municipality level explained 3% of the variability in 2005 and 2% in 2010. The model that best fit the data in both 2005 and 2010 included height, age, and sex at the child level; wealth index, location in an urban area, family size, and extended family at the household level; Gini coefficient and park density *z* scores and percentage of urban households at the municipality level; and the interaction terms. We eliminated caregiver education from all models because of multicollinearity with the wealth index.

**Table 2:** Hierarchical Linear Model of the Individual, Household, and Municipality Predictors of Body Mass Index *z* Score Relative to the World Health Organization Reference Population for Children and Adolescents, Colombia, 2005 and 2010^a^

Parameter	2005		2010	
	Unadjusted	Final	Unadjusted	Final
**Fixed effects^b^**				
Intercept, *y*_000_	−0.04	0.17^c^	−0.02	0.18
**Level 1 (child)**				
Height in cm, *y*_100_	—	0.01^d^	—	0.02^d^
Age in years, *y*_200_	—	−0.07^d^	—	−0.09^d^
Girl (vs boy), *y*_300_	—	0.17^d^	—	0.18^d^
**Level 2 (household)**				
Wealth index quintiles, *y*_010_	—	0.09^d^	—	0.13^d^
Urban (vs rural)^e^, *y*_020_	—	−0.09^d^	—	−0.09^d^
Family size (no. of members), *y*_030_	—	−0.05^d^	—	0.04^d^
Extended family (vs nuclear family), *y*_040_	—	0.05	—	0.05^c^
**Level 3 (municipality)**				
Wealth index, *y*_310_	—	−0.002	—	−0.02^c^
Gini coefficient (*z* score), *y*_001_	—	0.007	—	0.05
Park density (*z* score), *y*_002_	—	−0.02	—	0.02
Percentage urban^f^, *y*_003_	—	−0.002^c^	—	−0.002^c^
**Cross-level interactions**				
Height and wealth index, *y*_110_	—	0.01^d^	—	0
Age and wealth index, *y*_210_	—	−0.07^d^	—	−0.01^d^
Girl and wealth index, *y*_310_	—	−0.002	—	−0.02^c^
**Random parameters **				
Level 1 (child), *u*_00_	0.58^d^	0.57^d^	0.68^d^	0.67^d^
Level 2 (household), *r*_0_	0.41^d^	0.37^d^	0.32^d^	0.28^d^
Level 3 (municipality), σ^2^	0.03^d^	0.02^d^	0.02^d^	0.01^d^
**Model fit**				
Level 2 (household) intraclass coefficient	0.40	—	0.31	—
Level 2 (household) reliability	0.56	—	0.42	—
Level 3 (municipality) intraclass coefficient	0.03	—	0.02	—
Level 3 (municipality) reliability	0.46	—	0.53	—
Deviance	24,839	24,431	60,034	59,052
Akaike information criterion	24,847	24,473	60,042	59,094
Bayesian information criterion	24,875	24,623	60,074	59,261

Abbreviations: —, not applicable.

a Unless otherwise indicated, all values are beta coefficients. Sample weights were used to account for survey design.

b Regression coefficients should be interpreted as the average change in body mass index *z* score per unit change in the predictor, after controlling for all other covariates.

c *P* < .05.

d *P* < .01.

e A household is classified as urban if it is located in an area of the municipality with basic services, such as electricity, water, a town hall, etc. Otherwise, the household is classified as rural.

f Variable created by dividing the number of urban households in a municipality by the total number of households in the municipality.

### Three-level multinomial hierarchical linear model of weight status

For the underweight category, the unconditional model had a reliability of 0.15 in 2005 and 0.10 in 2010 at the household level and 0.46 and 0.48 at the municipality level (Table 3). At the child level for both years, girls were less likely than boys to be underweight, and the likelihood of underweight was inversely associated with height and positively associated with age. At the household level, the likelihood of underweight was inversely associated with the wealth index in 2005, and was higher in urban areas than in rural areas in 2010. Family size was a positive predictor of underweight in both 2005 and 2010. At the municipality level, the Gini coefficient *z* score was inversely associated with the likelihood of underweight in 2010 (ie, the greater the income inequality, the lower the likelihood of being underweight).

**Table 3:** Multinomial Hierarchical Linear Model of the Individual, Household, and Municipality Predictors of Underweight^a^ and Overweight^b^ Among Children and Adolescents, Colombia, 2005 and 2010^c^

Parameter	2005		2010	
	Unadjusted	Final	Unadjusted	Final
**Category 1 (Underweight^a^ vs Normal Weight)**				
**Fixed effects^d^**				
Intercept, *y*_000 _	0.21 (0.18–0.24)^e^	0.16 (0.12–0.22)^e^	0.22(0.21–0.24)^e^	0.21 (0.18–0.24)^e^
**Level 1 (child)**				
Height in cm, *y*_100_	—	0.98 (0.97–0.99)^e^	—	0.98 (0.97–0.99)^e^
Age in years, *y*_200_	—	1.12 (1.07–1.18)^e^	—	1.13 (1.09–1.17)^e^
Girl (vs boy), *y*_300_	—	0.70 (0.61–0.80)^e^	—	0.68 (0.62–0.74)
**Level 2 (household)**				
Reliability	0.15	—	0.10	—
Wealth index quintile, *y*_010_	—	0.92 (0.84–1.01)	—	0.90 (0.88–0.91)
Urban (vs rural)^f^, *y*_020_	—	1.28 (1.00–1.65)	—	1.21 (1.15–1.28)
Family size (no. of members), *y*_030_	—	1.06 (1.02–1.10)	—	1.04 (1.04.1.06)
Extended family (vs nuclear family), *y*_040_	—	0.86 (0.70–1.07)	—	0.96 (0.93–1.00)
**Level 3 (municipality)**				
Reliability	0.46	—	0.48	—
Gini coefficient *z* score, *y*_001_	—	1.02 (0.89–1.18)	—	0.92 (0.88–0.97)
Park density z score, *y*_002_	—	1.00 (0.90–1.11)	—	0.96 (0.92–1.00)
Percentage urban^g^, *y*_003_	—	1.01 (1.00–1.01)	—	1.00 (1.00–1.01)
**Category 2 (Overweight^b^ vs Normal Weight)**				
**Fixed effects^d^**				
Intercept, *y*_000_	0.17 (0.16–0.19)	0.27 (0.19–0.37)	0.19 (0.18–0.21)	0.27 (0.23–0.31)
**Level 1 (child)**				
Height in cm, *y*_100_	—	1.02 (1.01–1.04)	—	1.03 (1.03–1.04)
Age in years, *y*_200_	—	0.86 (0.81–0.93)	—	0.83 (0.80–0.86)
Girl (vs boy), *y*_300_	—	1.22 (1.04–1.42)	—	1.11 (1.02–1.21)
**Level 2 (household)**				
Reliability	0.16	—	0.09	—
Wealth index quintile, *y*_010_	—	1.21 (1.08–1.36)	—	1.28 (1.23–1.33)
Urban (vs rural)^f^, *y*_020_	—	1.04 (0.77–1.41)	—	0.9 (0.78–1.10)
Family size (no. of members), *y*_030_	—	0.90 (0.86–0.93)	—	0.92 (0.89–0.94)
Extended family (vs nuclear family), *y*_040_	—	0.99 (0.84–1.17)	—	1.13 (1.03–1.24)
**Level 3 (municipality)**				
Reliability	0.46	—	0.57	—
Gini coefficient *z* score, *y*_001_	—	1.07 (0.97–1.17)	—	1.05 (0.98–1.12)
Park density *z* score, *y*_002_	—	1.03 (0.92–1.15)	—	1.03 (0.98–1.09)
Percentage urban^g^, *y*_003_	—	1.00 (0.99–1.00)	—	1.00 (1.00–1.00)
**Random Parameters**				
**Variance intercept 1**				
Levels 1 and 2, r_0_	0.70	0.69^e^	0.49	0.48
Level 3, σ^2^	0.22^e^	0.19^e^	0.05^e^	0.04^e^
**Variance intercept 2**				
Levels 1 and 2, σ^2^	0.58^e^	0.86^e^	0.51	0.44
Level 3, *r*_0_	0.07^e^	0.05^e^	0.09^e^	0.04^e^

Abbreviations: —, not applicable.

a Underweight defined as more than 1 standard deviation below the mean.

b Overweight defined as more than 2 standard deviations above the mean.

c Sample weights were used to account for survey design.

d Regression coefficients are expressed as odds ratio (95% confidence interval) and should be interpreted as odds of being overweight or underweight compared with being normal weight for each predictor value or category after controlling for all other covariates.

e *P* < .01.

f A household is classified as urban if it is located in an area of the municipality with basic services, such as electricity, water, a town hall, etc. Otherwise, the household is classified as rural.

g Variable created by dividing the number of urban households in a municipality by the total number of households in the municipality.

For the overweight category, the unconditional model yielded a reliability of 0.16 at the household level and 0.46 at the municipality level in 2005 and 0.09 at the household level and 0.57 at the municipality level in 2010. At the child level, height and being a girl were positive predictors of the likelihood of overweight or obesity, and age was inversely associated. At the household level, the wealth index was positively associated with overweight, and being part of an extended family positively predicted the likelihood of overweight in 2010. The likelihood of overweight was inversely associated with family size. The Gini coefficient, park density, and percentage of urban households did not predict overweight.

## Discussion

In this study, we documented important contributions to variability in BMI among children and adolescents in Colombia: at the household level, wealth and being part of an extended family predicted the likelihood of a child being overweight or obese, and at the municipality level, income inequality was inversely associated with the likelihood of underweight. 

In Colombia, as in high-income countries, we found that girls were more likely to be overweight than boys. This outcome was expected because of a combination of social norms that encourage physical activity only among boys and the process of sexual maturation that increases body fat in girls (22). In contrast to what is observed in many high-income countries, we found that wealth was positively associated with overweight. Interestingly, the BMI gap between girls and boys in 2010 was significantly smaller among the wealthiest children and adolescents than among the poorest. As in high-income countries, girls from high socioeconomic strata in Colombia may be more concerned with weight control and may have more resources to achieve a healthy weight than girls from low socioeconomic strata. These interactions between sex and wealth suggest a need to design wealth- and sex-specific interventions that address underlying dynamics and prevent the burden of obesity from shifting to the poor.

The associations found at the household level may reflect particularities of the structure and tight bonds of Latin American families. Family size was inversely associated with overweight, consistent with evidence from high-income countries showing that having siblings and at least 2 adults in the household decreases the risk of overweight among children (8,23). Being part of an extended family was a positive predictor of overweight. Extended family members may influence the behavior of children and adolescents; for example, having a grandmother as the main caregiver may increase the likelihood of overweight in children (24). Older generations may still perceive undernutrition as a primary health problem and not recognize that overweight among children and adolescent is unhealthy (24). More information on the composition of these families, not currently available in national survey data, would be useful in understanding the conflicting associations of family size and extended family with overweight to inform the design of public health interventions.

Despite the small proportion of the variance in BMI *z* score clustered at the municipality level, 2 counterintuitive findings are worth mentioning. First, the Gini coefficient was inversely associated with underweight and positively associated with BMI *z* score (but not with overweight). The association between income inequality and BMI has been reported in high-income countries, but it is not common in low- and middle-income countries (25). Moreover, this association is usually linear, and it results in a greater prevalence of overweight in countries that have large income inequalities; it does not usually result in a lower prevalence of underweight, which we found in our study. The presence of wealthy community members may improve overall municipal infrastructure and increase access of economically disadvantaged households to health and social services (11). Another possibility is that factors at the municipality level that were not included in this model could explain these associations; substantial variance in BMI at the municipality level was unexplained. Studies examining the influence of income inequality at the community level and undernutrition at the individual level might explain these results. The second unexpected finding related to community-level predictors is the inverse association between urbanization and overweight. In high-income settings, urban areas are often considered to have limited opportunities for physical activity and healthy eating. Colombian children from urban households and communities may have better access to information, health services, and public policies promoting physical activity and preventing obesity, which are more common in Colombian urban areas. This association was significant only after controlling for wealth; because children and adolescents in cities are wealthier than those in rural areas, the association does not translate into a higher prevalence of overweight in rural areas.

This study has several limitations. It was an analysis of secondary data; hence, we could not assess the roles of predictors such as diet, physical activity, parental beliefs and behaviors, and details of family structure. A significant proportion of the variance at the individual level was unexplained after we adjusted for sex, age, and height. This variability could be explained by behavioral determinants of obesity that are not available in the database. The cross-sectional design limited our ability to establish causality. Finally, limited information at the municipality level decreased our sample size, and because the Colombian National Census is conducted every 10 years, information on park density and Gini coefficient at the municipality level from 2005 was used for the 2010 models. However, community-level predictors are relatively stable over time. Future studies should consider including historical information in studying the influence of community factors on individual outcomes.

A strength of this analysis was the use of an ecological model in combination with hierarchical linear models. Hierarchical linear models allow control for the clustering of the observations into different structures or organizations (such as families or municipalities) and also use clustering to assess the variability at each level of interaction and identify potential predictors (26).

We analyzed 2 nationally representative samples of Colombian children and adolescents and identified important individual, family, and community predictors of overweight. To our knowledge, this is the first study that used hierarchical linear models and a repeated cross-sectional design to study the influence of individual, family, and community factors on BMI in a nationally representative sample of children and adolescents. We identified important similarities and differences between children and adolescents in Colombia and in high-income countries. These factors should be considered when designing and implementing interventions to prevent obesity in children and adolescents. Results from this study could also be useful when designing and targeting interventions for other Latin American populations and other low- and middle-income countries that have characteristics similar to those of Colombia.
